# A multicentric evaluation of dipstick test for serodiagnosis of visceral leishmaniasis in India, Nepal, Sri Lanka, Brazil, Ethiopia and Spain

**DOI:** 10.1038/s41598-019-46283-9

**Published:** 2019-07-09

**Authors:** Sarfaraz Ahmad Ejazi, Sneha Ghosh, Samiran Saha, Somsubhra Thakur Choudhury, Anirban Bhattacharyya, Mitali Chatterjee, Krishna Pandey, V. N. R. Das, Pradeep Das, Mehebubar Rahaman, Rama Prosad Goswami, Keshav Rai, Basudha Khanal, Narayan Raj Bhattarai, Bhagya Deepachandi, Yamuna Deepani Siriwardana, Nadira D. Karunaweera, Maria Edileuza Felinto deBrito, Yara de Miranda Gomes, Mineo Nakazawa, Carlos Henrique Nery Costa, Emebet Adem, Arega Yeshanew, Roma Melkamu, Helina Fikre, Zewdu Hurissa, Ermias Diro, Eugenia Carrillo, Javier Moreno, Nahid Ali

**Affiliations:** 1 CSIR-Indian Institute of Chemical Biology, Kolkata, India; 2 Department of Biotechnology, Visva-Bharati, Santiniketan, West Bengal India; 3 Institute of Postgraduate Medical Education and Research, Kolkata, India; 4 Rajendra Memorial Research Institute of Medical Sciences, Patna, India; 5 School of Tropical Medicine, Kolkata, India; 6 B. P. Koirala Institute of Health Sciences, Dharan, Nepal; 7 Faculty of Medicine, University of Colombo, Colombo, Sri Lanka; 8 Aggeu Magalhhaes Institute, Oswaldo Cruz Foundation, Recife, Brazil; 9 Universidade Federal do Piaui, Teresina, Brazil; 10 University of Gondar, Gondar, Ethiopia; 11 Instituto de Salud Carlos III, Madrid, Spain

**Keywords:** Parasitic infection, Pathology

## Abstract

Visceral leishmaniasis (VL) is one of the leading infectious diseases affecting developing countries. Colloidal gold-based diagnostic tests are rapid tools to detect blood/serum antibodies for VL diagnosis. Lack of uniformity in the performance of these tests in different endemic regions is a hurdle in early disease diagnosis. This study is designed to validate a serum-based dipstick test in eight centres of six countries, India, Nepal, Sri Lanka, Brazil, Ethiopia and Spain with archived and fresh sera from 1003 subjects. The dipstick detects antibodies against *Leishmania donovani* membrane antigens (LAg). The overall sensitivity and specificity of the test with 95% confidence intervals were found to be 97.10% and 93.44%, respectively. The test showed good sensitivity and specificity in the Indian subcontinent (>95%). In Brazil, Ethiopia, and Spain the sensitivity and specificity of the dipstick test (83.78–100% and 79.06–100%) were better as compared to the earlier reports of the performance of rK39 rapid test in these regions. Interestingly, less cross-reactivity was found with the cutaneous form of the disease in Spain, Brazil, and Sri Lanka demonstrating 91.58% specificity. This dipstick test can therefore be a useful tool for diagnosing VL from other symptomatically similar diseases and against cutaneous form of leishmaniasis.

## Introduction

Visceral Leishmaniasis (VL) or kala-azar is one of the world’s most neglected tropical diseases second in mortality and fourth in morbidity (WHO Factsheet 2018). Ninety percent of the VL affected cases are from the endemic regions of Bangladesh, Nepal, India, Brazil, Ethiopia, and Sudan. Even though the numbers of VL cases have reduced, a sequel to VL, known as post Kala-azar dermal leishmaniasis (PKDL), has been emerging in high numbers and is considered a potential reservoir for infection^1,2^. Lack of reliable and accurate diagnosis and variations in responsiveness to different drugs are the major hurdles in disease management. Splenic or bone marrow aspiration and parasitological confirmation through microscopy is still the most prevalent test carried out to detect kala-azar^3,4^. However, these tests are not 100% sensitive as parasites may not be detected in the sample, which necessarily does not signify absence of the disease^5^. Subsequently immunofluorescent antibody test or IFAT^6^, direct agglutination test or DAT^7,8^ and ELISA (enzyme-linked immunosorbent assay) were developed to detect VL. Even though DAT and IFAT perform well in almost all endemic areas^9^ they show variations in the antigen preparations and high level of cross reactivity. They are also time consuming, entail expertise and need high-end instruments which restrict them to laboratories.

Therefore, these are a far cry from being a field adaptable diagnostic method for the endemic areas^10–12^. In all the high VL burden areas, a third generation of serodiagnosis emerged with rK39 recombinant antigen based rapid diagnostic tests (RDTs). Owing to its ease-of-use, stability, field adaptability and performance rK39 became the most used tool in diagnosis of the VL. Even though rK39 performs with high sensitivity and specificity especially in India, its performance varies with a sensitivity range from 36–100% and specificities from 90.8–100% in East Africa and Brazil^13,14^ (TDR 2011,WHO). The regional discrepancies in results led to manufacturing of RDT’s with local, region specific antigens for example rKE16, rK28, rKLO8^15–17^. These too have their drawbacks and are not the most preferred alternative to available diagnosis. Moreover, emerging PKDL cases are a challenge for the VL elimination program. There is no consensus on the use of serological tests for PKDL diagnosis since performance of the antigens show heterogeneity in several studies^10,18^. Besides, serological tests for VL diagnosis often showed cross reactivity with CL patients.

A pressing need for overcoming these shortcomings led us to develop a leishmanial membrane antigens (LAg) based serodiagnostic dipstick test which we showed 100% sensitive and specific against Indian and Brazilian VL and Indian PKDL^19^. Unlike whole parasite antigen used in DAT, LAg contains membrane antigens of promastigote thus it shows minimal cross reactivity with other diseases in comparison to broad antigen panel. With the severity of these diseases on the rise and the substantial variability in the results among various areas, it is a challenge to find a diagnostic test that can perform equivocally in all the regions. In the current study we aimed to determine the performance of this LAg-based diagnostic test in six global regions of VL endemicity and establish evidence of its specificity and sensitivity. Thus we report here a large scale global comparative study of a single immunochromatographic test across areas such as India, Nepal, Sri Lanka, Brazil, Ethiopia and Spain where VL is either prevalent or endemic.

## Results

**Figure 1:**
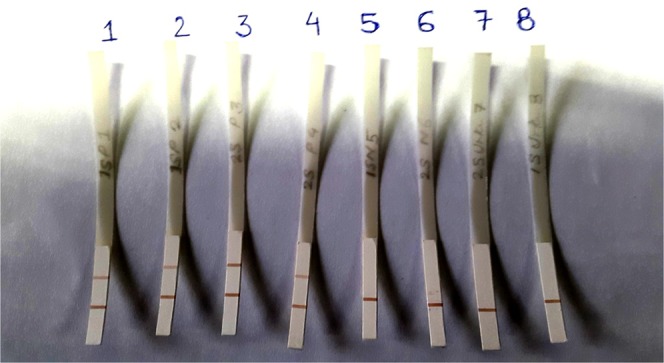
Representative picture of dipstick test with Indian kala-azar positive (1–4) and negative serum samples (5–8).

In this multicentric study a total of 1003 sera samples in six VL endemic regions across the globe were tested over a period of 4 years. Out of these, 463 were confirmed VL cases, 21 PKDL cases, 184 endemic healthy control, 105 non-endemic healthy control, 123 other symptomatically similar diseases and 107 CL cases (Table 1). The dipstick with two coloured bands at the test line and control line was considered positive and a single band at the control line was negative. A representative illustration of dipstick test is shown in Fig. 1”.

**Table 1:** Number of samples tested with dipstick and the obtained sensitivity and specificity in each region.

Countries	Centres	Kala Azar		PKDL		Sensitivity	NEHC		EHC		Other Diseases		CL		Specificity
		Pos	Neg	Pos	Neg		Pos	Neg	Pos	Neg	Pos	Neg	Pos	Neg	
	IICB	—	—	—	—		0	40	—	—	—	—	—	—	100
India	STM	69	1	10	0	98.75	—	—	0	6	0	15	—	—	100
	RMRIMS	140	3	11	0	98.05	0	5	1	37	2	12	—	—	94.73
	IPGMER	22	2	—	—	91.66	0	10	2	13	—	—	—	—	92
Nepal	BPKIHS	98	0	—	—	100	0	21	8	92	2	27	—	—	93.33
Sri Lanka	University of Colombo	3	2	—	—	60	0	13	—	—	1	53	9	69	93.10
Brazil	Universidade Federal do Piaui	40*	0	0	0	100	0	20*	—	—	—	—	0	20	100
Ethiopia	University of Gondar	86	0	—	—	100	0	16	5	11	4	7	—	—	79.06
Spain	Instituto de Salud Carlos III	31	6	—	—	83.78	—	—	0	9	—	—	0	9	100
Total		449	14	21	0	97.10	0	105	16	168	09	114	9	98	93.44

NEHC: Non-endemic healthy controls, EHC: Endemic healthy controls, CL: Cutaneous leishmaniasis; *Data obtained in previous study^19^.

### India

The blind test was conducted with archived serum samples of 143 confirmed VL cases, 11 PKDL and 57 control samples at Rajendra Memorial Research Institute of Medical Sciences (RMRIMS), Patna during March 2013 to June 2013. The tests performed well and the results of these Indian samples showed sensitivity of 98.05% for VL (140/143) and PKDL (11/11) cases. Out of 140 dipstick positive VL cases one sample was false negative with rK39 and two out of three false negative found in dipstick test were positive with rK39 test. Of the 57 control samples one endemic healthy control and 2 other diseases showed cross reactivity in dipstick test, thereby depicting an overall 94.73% (54/57) specificity in that centre.

Blind folded dipstick test was carried out at the Institute of Postgraduate Medical Education and Research (IPGMER) in Kolkata on 25 archived VL cases and 25 healthy controls (endemic and non-endemic) confirmed by rK39 test. One VL case went invalid in dipstick test. Out of 24 VL cases two cases showed false negative results in the dipstick test thus provided 91.66% (22/24) sensitivity. Two endemic healthy control samples gave false positive result in the dipstick test thus showed 92% (23/25) specificity.

We collected fresh blood samples from patients having symptoms of VL and were confirmed parasitologically at School of Tropical Medicine (STM), Kolkata. Of the samples tested, 69 out of 70 VL cases were detected in the dipstick test along with 10 PKDL cases with overall sensitivity of 98.75%. Interestingly, there have been instances (n = 5) where parasitologically confirmed VL cases showed negative results with rK39 strip test but tested positive with PCR and dipstick test. Six endemic healthy controls and 15 other diseases collected from STM showed no cross reactivity in dipstick test. Forty samples from non-endemic healthy controls were collected and tested in the Indian Institute of Chemical Biology (IICB), Kolkata with 100% specificity.

### Nepal

**Figure 2:**
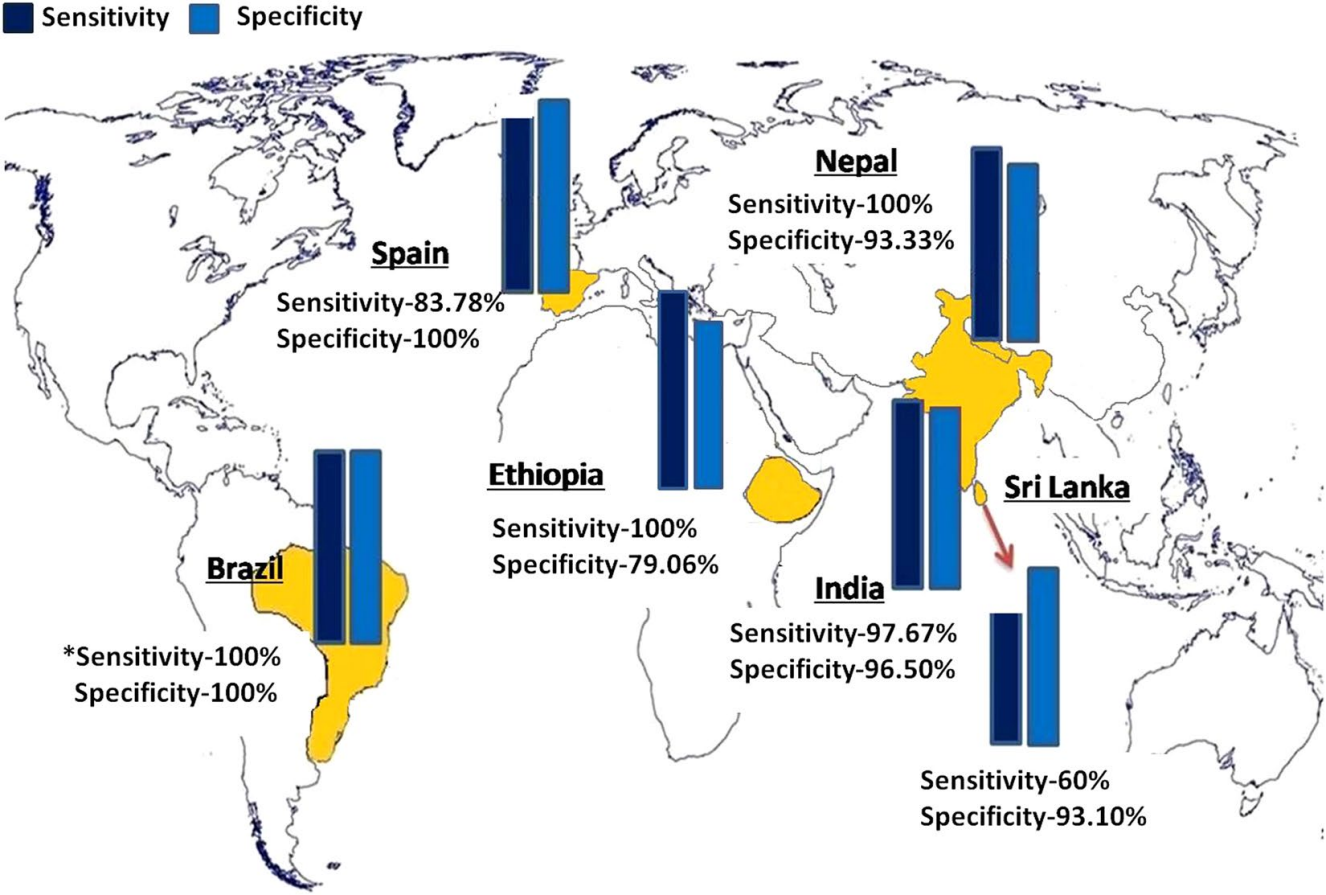
Performance of dipstick test in six countries, India, Nepal, Sri Lanka, Brazil, Ethiopia and Spain. Sensitivity in Brazil depicted from previous study (*)^19^.

To evaluate this diagnostic dipstick test in the endemic region of Nepal a blind folded test was carried out at the B.P. Koirala Institute of Health Sciences, Dharan, Nepal. A total of 248 tests were conducted, 98 confirmed VL cases, 100 endemic healthy controls, 21 non-endemic healthy control and 29 other diseases which were pathologically confirmed. The sensitivity of the test in Nepal was found to be 100% (98/98) without any false negative results (Fig. 2). The overall specificity of the test including all 150 controls was 93.33% (140/150). Eight endemic healthy controls and two other diseases showed cross reactivity in the dipstick test.

### Sri Lanka

Striking results were obtained from samples tested at University of Colombo, Sri Lanka. Out of 5 cases of autocanthous VL in Sri Lanka, dipstick showed positivity with 3 serum samples. Interestingly, in Sri Lanka, both VL and CL are caused by *L*. *donovani* infection. On testing 78 such CL samples our dipstick could identify 69 of them as negative therefore making our test highly specific for VL even if the causative agent is the same. With a total of 67 control samples including 13 non-endemic healthy controls, 14 other symptomatically similar diseases to VL such as malaria, viral fever, typhoid, pneumonia, etc. and 40 other skin diseases, dipstick showed cross reactivity with only one sample demonstrating an overall 98.50% (66/67) specificity of the test. However, including tested CL cases in the calculation showed 93.10% (135/145) specificity overall.

### Brazil

Our group had previously reported (Saha *et.al*) 100% sensitivity and specificity of dipstick test on 60 samples from Brazil (40 VL and 20 healthy controls). We in this study additionally tested 20 CL cases in Brazil infected with *L*. *braziliensis*. Brazil is amongst the seven countries reporting 90% CL cases^20^. None of the CL samples showed cross reactivity with *L*. *donovani* antigen, LAg, in the dipstick test. Thus dipstick did not recognize tegumentary form of leishmaniasis in Brazil.

### Ethiopia

A blind-fold study was conducted at the University of Gondar, Ethiopia. A total of 129 samples were tested, out of which 86 were acute VL and 43 control samples including 11 other diseases, 16 endemic healthy controls and 16 non-endemic healthy controls. VL cases were confirmed with bone marrow (n = 21) or splenic aspirates (n = 65). Our dipstick in Ethiopia showed overall 100% sensitivity and 79.06% specificity. Interestingly, one confirmed VL case was positive in our dipstick but negative with rK39 strip test. Out of eleven other diseases four samples of pneumonia, spontaneous bacterial peritonitis, abscess and one unknown disease showed positive results with the dipsticks. All the 16 non-endemic controls were found negative with the dipsticks. However, in the endemic healthy controls, 5 out of 16 were false positive. rK39 test could not be performed with the other diseases and endemic healthy controls to establish the serological status of the cases.

### Spain

A total of 55 serum samples were tested in Instituto de Salud Carlos III, Madrid, Spain, including 37 samples of confirmed VL cases, 9 endemic healthy controls and 9 CL samples. VL cases were confirmed with serological test such as ELISA and IFI. PCR was done with bone marrow and peripheral blood for VL and skin biopsy for CL. Our dipstick tests showed positive results in 31 out of 37 confirmed VL cases thus showing 83.78% sensitivity. Two confirmed and dipstick positive VL cases showed negative result with IFI titre less than 1/40 and four VL cases with antibody titre less than 0.2 in ELISA. Seventeen dipstick positive confirmed VL cases were negative in PCR while eight dipstick positive VL cases were negative with rK39 strip test. However, two confirmed VL cases which were negative in our dipstick test were found to be positive with rK39 strip test. All the 9 endemic control samples were found negative in dipstick test and also in PCR and rK39 strip test. The dipstick did not show any cross reactivity with any of the nine CL samples. However, one serum sample in PCR and two in rK39 test showed cross reactivity with CL.

## Discussion

Herein, we report the performance of LAg, a leishmanial membrane extract, as a diagnostic marker in a dipstick format and validated this across six VL endemic countries for global comparative evaluation. Our LAg-based dipsticks were distributed amongst our collaborators situated in various VL endemic regions and unbiased test against 1003 samples was done for validation. Our findings illustrate a high sensitivity (97.10%) and specificity (93.44%) of the test in all these regions collectively.

A range of techniques are available to detect VL across the world out of which microscopy of tissue aspirates such as spleen and bone marrow are still the gold standard test^21^. However, these are invasive, have variable sensitivities, technician dependent and complex. Apart from microscopy several tests are available in the form of molecular diagnostics like PCR, serological tests such as DAT and IFAT, or rapid diagnostic tests like rK39. Each of these has its own advantages and disadvantages and discrepancy in results. Because of which none has been able to withstand as a standalone test for VL alone. DAT which uses whole parasite antigen has proved to be highly specific and sensitive in most of the endemic areas, and certain areas where HIV co-infection is prevalent for example in Spain and other parts of Europe. In fact a recent report claims the performance of DAT to be better than rK39 based RDTs when tested against archived samples in Spain^5^, moreover, it is the most cost effective test in Brazil^9^. Recently a multicentre validation of DAT showed sensitivity ranging from 96·2–99·5% and specificity between 96·2–97·5%^22^. However, due to its cumbersome process and high levels of cross reactivity with other trypanosomatids, rK39 based tests are preferred over DAT. rK39 recombinant antigen based diagnosis is a widely used RDT as it is easy to use, is highly sensitive for samples in the Indian subcontinent, and is stable. However, its variability in performance between regions is an area of concern especially in East Africa where its sensitivity is reported to be as low as 67.6%^5^. Our focus was therefore to make a dipstick test with the leishmanial membrane antigens (LAg), with the advantages of high sensitivity of DAT, along with the ease of use and robustness of RDTs like rK39.

For diagnosis of VL, rK39 RDT has been very well studied in the Indian subcontinent where WHO recommended its use and its inclusion in national VL elimination program for diagnosis^23^. We previously tested our LAg-based dipstick in India with 100% sensitivity and specificity^19^. To further validate, we tested samples across the highly endemic regions of India that is Bihar and West Bengal. 97.67% (252/258) sensitivities and 96.50% (138/143) specificities were obtained at the four centres in India. Based on the study in India performance of dipstick is comparable with rK39 in this region.

Over the years of this study we and others^18^ have observed a striking increase in PKDL cases in India, which many a time gets confused with leprosy thus delaying treatment. Patients suspected with PKDL are usually tested by slit-skin smear or skin biopsy, which may fail to show the presence of parasites in lesions^18^. Since PKDL is a reservoir for parasites, and could be the cause behind newer cases, it has become imperative for any diagnostic test to be able to determine these cases. Herein our dipstick showed 100% (21/21) sensitivity in detecting PKDL cases in India.

A pertinent problem with VL cases in Nepal is the delay in detection of the disease from the inception of symptoms^24^. Even though the number of VL cases in Nepal is decelerating, new VL cases have been reported over the past years in areas which were previously non-endemic^25^. LAg dipstick herein performs very well amongst all the tested samples with 100% sensitivity. Amongst the control tests, non-endemic healthy controls were detected with 100% specificity. However, cross reactivity amongst the endemic healthy controls and other diseases resulting in overall specificity of 93.33%.

Leishmaniasis in Sri Lanka is a newly emerging disease and established as a notifiable public health problem only after 2008^26^. *L*. *donovani*, otherwise known to cause VL in East Africa and India, is the causative agent for CL in Sri Lanka^27,28^. We were therefore very interested in testing for any cross reactivity of our dipstick, which is coated with *L*. *donovani* membrane antigen. Interestingly, our dipstick test show negative results for such CL cases in 69 out of 78 samples. Moreover, with very few VL samples tested in Sri Lanka dipstick showed positive result with three out of five Sri Lankan VL tested.

Brazil has been reported with around 0.2 million VL cases and 0.7–1 million CL cases. Therefore it is extremely important for a diagnostic test to be able to distinctly detect VL which should not show cross reactivity especially with tegumentary leishmaniasis. We have previously shown 100 percent sensitivity and specificity of our LAg-dipstick in detecting VL from Brazilian serum samples^19^. Other than microscopy, CL has been attempted to be diagnosed with RDTs like rK39, but results show a lot of variability from 10–100% specificity^29,30^. We therefore checked if our dipstick test could differentiate CL from VL cases and found no cross reactivity of dipstick test with CL samples in Brazil.

Eastern African countries are the third most endemic areas for VL especially Ethiopia, Sudan and South Sudan. Like in the other endemic regions, here too, parasite detection along with DAT or rK39 is the diagnostic regime. Due to low level of accuracy of rK39 in these regions, local antigens like rKLO8, or newer antigens like rk28 have been tested. To our delight, in our study we found 100% sensitivity and 79.06% specificity of dipstick test in Ethiopia.

A study conducted in Spain with our LAg-based dipsticks also fared well with good sensitivity and specificity. With limited number of samples the dipstick test showed its best performance in detecting VL cases when compared individually with other reference tests, IFI, ELISA and PCR. Dipstick was also found better in discriminating CL cases than PCR and rK39 test. In a recent study, it was reported that DAT performs better than rk39 in samples co-infected with HIV. This could be due to the whole antigen used in DAT^5^. As we also utilize specific leishmanial membrane extracted antigen, it is possible that our dipstick can perform well amongst the HIV co-infected patients also, which would be an interesting study for future. In the current study antigen LAg was used in a dipstick format that can be applied in field settings with limitations of serum dilution and washing steps. To make the dipstick fully field adaptable we have transformed it into the lateral flow based rapid test which will be validated in the near future.

As none of tests alone is enough to confirm VL, it is suggested that two confirmatory tests be performed^11^. This study deals in the global comparison of a single test across major VL affected areas. The results are highly encouraging and since this study utilises the best of both DAT and RDTs it can be a single cost effective confirmatory test for detection of VL in the endemic areas.

## Methods

### Study design

We aimed to test the performance of leishmanial antigens, LAg in the dipstick format across several VL endemic regions worldwide, thereby chose participants from eight VL endemic centres comprised of six different countries where 1003 samples were tested.

### Antigen preparation

*L*. *donovani* membrane antigens (LAg) were prepared from cell pellets of *L*. *donovani* promastigote strain (ATCC^®^ PRA-413™)^19,31^. In brief, cell pellets were washed three times in 0.02 M PBS and then suspended in hypotonic medium of 5 mM cold Tris-HCl buffer, pH 7.6 (1 g of cell pellet in 50 ml of 5 mM Tris–HCl). Cells were then vortexed vigorously (6 times for 2 min each with 10 min intervals in ice) to make the membrane surface leaky and effective release of all cytoplasmic matrix from the cells in the hypotonic medium. Following this, the suspension was centrifuged (2310 × g for 10 min at 4 °C) (Biofuge^TM^Stratos^TM^, Heraeus, Germany) to obtain the ghost membrane as pellet containing membrane bound proteins. Pellets along with 10 ml of ice-cold Tris-HCl buffer were then sonicated with 6 cycles of 30 sec pulse and 1 min interval at 4 °C. Sonication dissociates the protein from the membrane and was collected in the supernatant by centrifugation (5190 × g, at 4 °C for 30 min). Protein concentration of LAg was estimated by the Lowry’s method. A particular pattern of predominant proteins of LAg was ensured through SDS-PAGE before the dipstick preparations^32^.

### Dipstick development and assay

Dipsticks for the assay were prepared following previous protocol as mentioned by Saha *et.al*.^19^ Briefly, LAg (1.0 µg/4 mm dipstick) at the test line and rabbit anti-human IgG (1:10 dilution) at control line were coated onto a nitrocellulose membrane (8 cm × 2.4 cm). Further the membrane was blocked overnight with 2% BSA + 0.1% Tween-20 and 0.01% NaN_3_ in 100 mM Tris Buffer Saline (TBS) at 4 °C which was followed by washing with TBS + 0.05% Tween-20 (TBST), drying and adhering to the moisture unreceptive plastic sheet, cut and stored at RT in desiccation until the test.

The assay comprises of incubation of the dipstick with diluted serum samples (1:2000) for 30 mins which is followed by washing with TBST (twice). Subsequently, it is incubated with HRP conjugated anti-human IgG (1: 2000) for 30 mins. Finally, after two washes in TBST and one in TBS, the strips are dipped in a freshly prepared substrate composed of 0.05% 3, 3′-diaminobenzidine tetrahydrochloride (DAB, Sigma, USA) containing 0.05% of H_2_O_2_ in 100 mM TBS. The reaction is stopped by dipping in distilled water. The appearance of dark brown coloured bands at both the test and control line indicates VL positivity and a single band at the control line is indicative of VL negativity.

### Assay selection

Freshly prepared batches of strips quality tested with confirmed positive and confirmed negative VL cases were shipped to each laboratory clearly mentioning the storage conditions and shelf-life of the dipsticks. Each lab was sent a written protocol and a video of the test being performed by us for uniformity in conducting the test. Each laboratory assembled a performance panel using locally archived and depersonalized sera. VL was confirmed parasitologically (from spleen, bone marrow, or lymph node) by microscopy and/or culture. Healthy endemic control samples and potentially cross reactive samples were also collected and tested. The samples used were unknown/blind for the performer at the time of the assay which was disclosed after the test completion and interpretation.

### Result interpretation

Test results were analyzed, interpreted and recorded blindly by at least two observers on standardized form from all participating regions. The final results were compiled and analysed by us to determine the sensitivity and specificity of the dipstick test in each region.

### Ethical statement

This study was approved by the Human Ethics committee of CSIR-Indian Institute of Chemical Biology. Additionally, each participating laboratory obtained local or institutional ethical clearance for carrying out the tests. Written informed consents were taken from each participant and methods performed in this study were according to the ethical guidelines.

## Data Availability

All data generated or analyzed during this study are included in this published article.
