# Author Correction: A multicentric evaluation of dipstick test for serodiagnosis of visceral leishmaniasis in India, Nepal, Sri Lanka, Brazil, Ethiopia and Spain

**DOI:** 10.1038/s41598-021-83332-8

**Published:** 2021-02-11

**Authors:** Sarfaraz Ahmad Ejazi, Sneha Ghosh, Samiran Saha, Somsubhra Thakur Choudhury, Anirban Bhattacharyya, Mitali Chatterjee, Krishna Pandey, V. N. R. Das, Pradeep Das, Mehebubar Rahaman, Rama Prosad Goswami, Keshav Rai, Basudha Khanal, Narayan Raj Bhattarai, Bhagya Deepachandi, Yamuna Deepani Siriwardana, Nadira D. Karunaweera, Maria Edileuza Felinto deBrito, Yara de Miranda Gomes, Mineo Nakazawa, Carlos Henrique Nery Costa, Emebet Adem, Arega Yeshanew, Roma Melkamu, Helina Fikre, Zewdu Hurissa, Ermias Diro, Eugenia Carrillo, Javier Moreno, Nahid Ali

**Affiliations:** 1 CSIR-Indian Institute of Chemical Biology, Kolkata, India; 2 Department of Biotechnology, Visva-Bharati, Santiniketan, West Bengal India; 3 Institute of Postgraduate Medical Education and Research, Kolkata, India; 4 Rajendra Memorial Research Institute of Medical Sciences, Patna, India; 5 School of Tropical Medicine, Kolkata, India; 6 B. P. Koirala Institute of Health Sciences, Dharan, Nepal; 7 Faculty of Medicine, University of Colombo, Colombo, Sri Lanka; 8 Aggeu Magalhhaes Institute, Oswaldo Cruz Foundation, Recife, Brazil; 9 Universidade Federal do Piaui, Teresina, Brazil; 10 University of Gondar, Gondar, Ethiopia; 11 Instituto de Salud Carlos III, Madrid, Spain

Correction to: *Scientific Reports* https://doi.org/10.1038/s41598-019-46283-9, published online 09 July 2019

The original version of this Article contained an error.

The information in the “Dipstick development and assay” section was incomplete. Therefore, the original text,

“The assay comprises of incubation of the dipstick with diluted serum samples (1:2000) for 30 min which is followed by washing with TBST (twice). Subsequently, it is incubated with HRP conjugated anti-human IgG (1: 2000) for 30 min. Finally, after two washes in TBST and one in TBS, the strips are dipped in a freshly prepared substrate composed of 0.05% 3, 3′-diaminobenzidine tetrahydrochloride (DAB, Sigma, USA) containing 0.05% of H_2_O_2_ in 100 mM TBS. The reaction is stopped by dipping in distilled water. The appearance of dark brown coloured bands at both the test and control line indicates VL positivity and a single band at the control line is indicative of VL negativity.”

now reads:

“The general assay consists of incubation of the dipsticks with diluted serum (1:2000) samples for 30 min, followed by washing with TBST (twice). Subsequently, it is incubated with HRP conjugated anti-human IgG (1:2000) for 30 min. Finally, after two washes in TBST and one in TBS, the strips are dipped in a freshly prepared substrate composed of 0.05% 3, 3′-diaminobenzidine tetrahydrochloride (DAB, Sigma, USA), containing 0.05% of H_2_O_2_ in 100 mM TBS. The reaction is stopped by dipping in distilled water, and the strips dried at RT. In Spain, there were several modifications to the assay; the dipsticks are incubated in diluted serum (1:100), and HRP conjugated anti-human IgG, for 60 min, followed by a 10 min incubation in substrate composed of 0.07% of DAB and 0.2% of H_2_O_2_ in 60 mM TBS, before being dried at RT. The appearance of dark brown coloured bands at both the test and control line indicates VL positivity and a single band at the control line is indicative of VL negativity. Whenever this signal was hard to assess, we classified it as not clear, and therefore grouped it together with those displaying no bands. Dipsticks that did not present with a reactive band, or a control band, were excluded from further consideration.”

These changes do not affect the overall conclusions of the Article. This has now been corrected in the PDF and HTML versions of the Article.

